# Improvement of hydrogen production from *Chlorella* sp. biomass by acid-thermal pretreatment

**DOI:** 10.7717/peerj.6637

**Published:** 2019-03-21

**Authors:** Tran T. Giang, Siriporn Lunprom, Qiang Liao, Alissara Reungsang, Apilak Salakkam

**Affiliations:** 1 Department of Biotechnology, Faculty of Technology, Khon Kaen University, Khon Kaen, Thailand; 2 Research Group for Development of Microbial Hydrogen Production Process from Biomass, Khon Kaen University, Khon Kaen, Thailand; 3 Key Laboratory of Low-grade Energy Utilization Technologies and Systems, Chongqing University, Ministry of Education, Chongqing, China; 4 Institute of Engineering Thermophysics, Chongqing University, Chongqing, China

**Keywords:** Physico-chemical pretreatment, Microalgal biomass, Renewable energy, Anaerobic digestion, Dark fermentation, Third generation biofuel

## Abstract

**Background:**

Owing to the high growth rate, high protein and carbohydrate contents, and an ability to grow autotrophically, microalgal biomass is regarded as a promising feedstock for fermentative hydrogen production. However, the rigid cell wall of microalgae impedes efficient hydrolysis of the biomass, resulting in low availability of assimilable nutrients and, consequently, low hydrogen production. Therefore, pretreatment of the biomass is necessary in order to achieve higher hydrogen yield (HY). In the present study, acid-thermal pretreatment of *Chlorella* sp. biomass was investigated. Conditions for the pretreatment, as well as those for hydrogen production from the pretreated biomass, were optimized. Acid pretreatment was also conducted for comparison.

**Results:**

Under optimum conditions (0.75% (v/v) H_2_SO_4_, 160 °C, 30 min, and 40 g-biomass/L), acid-thermal pretreatment yielded 151.8 mg-reducing-sugar/g-biomass. This was around 15 times that obtained from the acid pretreatment under optimum conditions (4% (v/v) H_2_SO_4_, 150 min, and 40 g-biomass/L). Fermentation of the acid-thermal pretreated biomass gave 1,079 mL-H_2_/L, with a HY of 54.0 mL-H_2_/g-volatile-solids (VS), while only 394 mL/L and 26.3 mL-H_2_/g-VS were obtained from the acid-pretreated biomass.

**Conclusions:**

Acid-thermal pretreatment was effective in solubilizing the biomass of *Chlorella* sp. Heat exerted synergistic effect with acid to release nutrients from the biomass. Satisfactory HY obtained with the acid-thermal pretreated biomass demonstrates that this pretreatment method was effective, and that it should be implemented to achieve high HY.

## Introduction

The demand for energy has been rising accompanying the expansion of industrial world, with fossil fuels, e.g., gasoline and diesel fuel, being the primary energy sources. At a current consumption rate, it has been projected that the supply of these energy sources will be short of demand in the near future (Bundhoo & Mohee, 2016). The use of fossil fuels also releases greenhouse gasses, contributing to global warming. Considering these, it is necessary that alternative energy resources that can be produced in a large quantity and are environmentally friendly are used in place of fossil fuels (Choi et al., 2016). On the basis of production potential, energy content, and combustion ability, hydrogen is among the promising alternative energy sources worth the attention and use (Matsakas et al., 2017; Møller et al., 2017).

Hydrogen can be produced by fermentation of sugar- and starch-containing materials. In the past decades, microalgal biomass, regarded as the feedstock of third generation, has emerged as a high potential feedstock for hydrogen production (Chen et al., 2013). This is due to, for example, its short production cycle, low water demand, no competitive uses with food and feed production, and high contents of fermentable nutrients (Simas-Rodrigues et al., 2015). Hydrogen production from microalgal biomass (*Chlorella vulgaris*) has been reported to be as high as 135 mL/g-volatile-solids (VS) (Wieczorek, Kucuker & Kuchta, 2014). However, according to our previous reports (Phanduang et al., 2017; Lunprom et al., 2019), the practical hydrogen yield (HY) obtained from microalgal biomass (*Chlorella* sp.) is far from the stoichiometrically maximum. This was considered due partly to rigid and difficult-to-degrade cell wall of the microalgae as suggested by Tijani, Abdullah & Yuzir (2015) and He, Dai & Wu (2016).

In order to enhance the fermentation yield from microalgal biomass, disruption of microalgal cells is necessary (Xia et al., 2015), and therefore several pretreatment methods have been applied to the biomass (Velazquez-Lucio et al., 2018; Wang & Yin, 2018). However, no method is accepted as a general method since microalgal characteristics are diverse, making pretreatment methods and conditions differ from strain to strain. Chemical pretreatment is usually preferred to other methods because it offers higher conversion efficiency of biomass to simple sugars (Roy et al., 2014). Among all known chemical pretreatment methods, dilute acid hydrolysis is probably the most widely used method. This is because acids can effectively solubilize polysaccharides (Tomás-Pejó et al., 2011). It causes swelling of organic structure, making it more vulnerable to hydrolysis (Yun et al., 2013). Acid pretreatment can be performed at room temperature. However, it is usually accomplished with a combination with heat (Quintero, Rincón & Cardona, 2011; Argun, Gokfi & Karapinar, 2016). Acid-thermal pretreatment was shown to be effective in pretreating various types of microalgal biomass, including *Chlorella* spp. biomass (Ho et al., 2013; Chen, Chang & Chang, 2016), *Scenedesmus* spp. biomass (Kondaveeti et al., 2014), and *Dunaliella* sp. biomass (Karatay et al., 2016). Example of acids used include H_2_SO_4_, HCl, H_3_PO_4_, and HNO_3_ (Argun, Gokfi & Karapinar, 2016). H_2_SO_4_ was shown to be effective in hydrolyzing not only hemicellulose (Schell et al., 2003), but also the biomass of *Chlorella* sp. to soluble sugars for the production of ethanol (Ho et al., 2013), and hydrogen (Chen et al., 2014). HCl was used successfully at 1.2% (v/w) to hydrolyze *Chlorella* sp. biomass, producing 37 mL-H_2_/g-DW (Yun et al., 2013). Choi et al. (2016) used response surface methodology (RSM) to optimize a combined (acid and thermal) pretreatment conditions to enhance hydrogen production from biomass of *C. vulgaris*. The maximum yield of 48.4 mL-H_2_/g-dry cell weight (DCW) was obtained under the optimum conditions of 1.0% HCl, 92 °C, and pretreatment time of 47 min.

In this study, acid-thermal pretreatment was applied to biomass of *Chlorella* sp. in order to release fermentable sugars to facilitate hydrogen production. Conditions for the pretreatment, i.e., acid species, acid concentration, pretreatment time, and biomass concentration, were optimized. In addition, to ensure maximum hydrogen production from the pretreated biomass, conditions for dark fermentation of pretreated biomass were optimized. Acid pretreatment was also conducted as another treatment in the present study for a comparison with the acid-thermal method. The efficiency and effect of the pretreatment methods are demonstrated through the reducing sugar yield, pretreatment efficiency, and hydrogen production.

## Materials & Methods

### Substrate and inoculum

*Chlorella* sp. was grown on coal-fixed flue gas at Fuqing King Dnarmsa Spirulina Co. Ltd., Fujian, China, and was supplied in the form of dry powder. It was stored in an air-tight bucket at −20 °C until use. The biomass contained (by weight) 52.3 ± 0.3% protein, 29.2 ± 0.4% carbohydrate, 8.7 ± 0.1% fat, 5.1  ± 0.0% ash, and 4.7  ± 0.0% moisture. Ultimate analysis revealed that the biomass contained 47.2  ± 0.2% carbon, 6.5 ± 0.0% hydrogen, 30.2 ± 0.1% oxygen, 8.4 ± 0.1% nitrogen, and 0.6 ± 0.0 sulfur. The empirical formula of the biomass, excluding sulfur, was C_6.55_H_10.83_O_3.15_N. The carbon to nitrogen (C/N) ratio of the biomass was 5.6.

Anaerobic granular sludge of an anaerobic digester was used as an inoculum to produce hydrogen. It was provided by Khon Kaen Brewery Co., Ltd., Khon Kaen, Thailand, and stored at 4 °C until use. After being heat-treated at 105 °C for 4 h, the granules were acclimatized in a modified basic anaerobic (BA) medium containing 10 g/L of *Chlorella* sp. biomass. The granules were transferred to a fresh medium every three days for four cycles before use. The modified BA medium was prepared following Fangkum & Reungsang (2011).

### Optimization of acid and acid-thermal pretreatment conditions

For acid pretreatment, four factors, i.e., type of acid, acid concentration, biomass concentration, and pretreatment time, were optimized. Four acids, i.e., HCl, H_2_SO_4_, HNO_3_, and H_3_PO_4_ were used. The biomass was suspended in 3% (v/v) acid solutions at 30 g-dry-weight (DW)/L. Then, the suspensions were left at 35 ± 3 °C for 30 min at 150 rpm. The acid that gave the highest reducing sugar yield and pretreatment efficiency was selected for use in the subsequent optimization of acid concentration in a range of 0.5–10% (v/v). Pretreatment efficiency was defined as the ratio of the reducing sugar yield (g-reducing-sugar/g-DW) to inhibitor concentration (g-inhibitor/g-DW). Biomass concentration was optimized next by varying the concentration from 10 to 50 g/L. Then, pretreatment time (15–180 min) was optimized. Distilled water was used in place of acid in the control experiment.

As for the optimization of acid-thermal pretreatment conditions, the concentration of the selected acid was firstly optimized over the range 0–5% (v/v), with the use of pretreatment temperature, time and biomass concentration of 120 °C, 30 min, and 30 g/L, respectively. Then, the pretreatment temperature (110–200 °C), pretreatment time (0–60 min), and biomass concentration (10–50 g/L) were optimized. The heating apparatus was an 11 L oil bath (WiseBath WHB-11; DAIHAN SCIENTIFIC CO., LTD. Daihan Scientific Co., Wonju, South Korea) containing 5 L of silicone-based oil (JULABO, Seelbach, Germany). Biomass suspension was transferred into a Teflon-lined 150 mL stainless steel vessel before being immersed in the oil bath, which was pre-heated to the desired temperature. The vessel was left in the oil bath for the desired time before being removed and immediately cooled in an ice bath. After the pretreatment, the pretreated slurries were neutralized by adding 10 M NaOH to raise the pH to around 6.0.

### Optimization of hydrogen production from acid and acid-thermal pretreated biomass

Hydrogen fermentations were conducted using the whole pretreated slurries from the acid and acid-thermal pretreatments. The conditions for the fermentations were optimized by varying the substrate concentration from 5 to 25 g-VS/L for the acid pretreated biomass, and 5 to 35 g-VS/L for the acid-thermal pretreated biomass. Next, the substrate to inoculum (S/I) ratio was varied from 1.0 to 5.0 on a VS basis. Then, initial pH value (4.0–7.0) was optimized. The fermentations were conducted in 60-mL serum bottles with a working volume of 35 mL. The headspace was flushed with nitrogen gas for 10 min to create anaerobic conditions. No reducing agents were used to maintain the anaerobic conditions as the anaerobic granules used in the present study contained facultative anaerobes (Nualsri, Kongjan & Reungsang, 2016), which could consume oxygen, maintaining anaerobic conditions. Incubation was carried out at 35 ± 3 °C and 150 rpm. The volume of biogas was periodically measured using a wetted glass syringe. Gas samples were collected using a gas-tight syringe. All the experiments were conducted in triplicate and the average values are reported with their standard deviations.

### Microbial community analysis

Polymerase chain reaction-denaturing gel gradient electrophoresis (PCR-DGGE) was used to analyze the microbial community during the late hydrogen production phase. The analysis was conducted using the method of Jehlee et al. (2017).

### Analytical methods

Hydrogen content in the biogas was analyzed using gas chromatography (GC) following the method of Sitthikitpanya et al. (2017). The values of the kinetic parameters for hydrogen production were estimated using the modified Gompertz equation as decribed by Khanal et al. (2004). Volume of hydrogen gas (mL) was calculated using an equation proposed by Zheng & Yu (2005). The composition of *Chlorella* biomass was determined using standard methods at the Food Research and Testing Laboratory (FRTL), Faculty of Science, Chulalongkorn University, Thailand. Elemental composition of the biomass was analyzed using a CHNS-O Analyzer (Flash EA 1112; Thermo Quest, Milan, Italy) at the Scientific Equipment Center, Prince of Songkla University, Thailand. Reducing sugar concentration was determined using the DNS method (Miller, 1959) with glucose as a standard. The concentration of inhibitors in the form of furans (5-hydroxymethylfurfural (HMF) and furfural) was determined spectrophotometrically following the methods of Martinez et al. (2000) and De Andrade et al. (2017). Volatile fatty acids (VFAs) concentrations were analyzed following the method of Nualsri, Kongjan & Reungsang (2016). The morphology of the microalgal cells before and after the pretreatments was examined under a scanning electron microscope (SEM) (model JSM-6610LV, JEOL Ltd., USA) at the Scientific and Technological Research Equipment Centre, Chulalongkorn University, Thailand.

## Results

### Optimization of acid and acid-thermal pretreatment conditions

**Figure 1:**
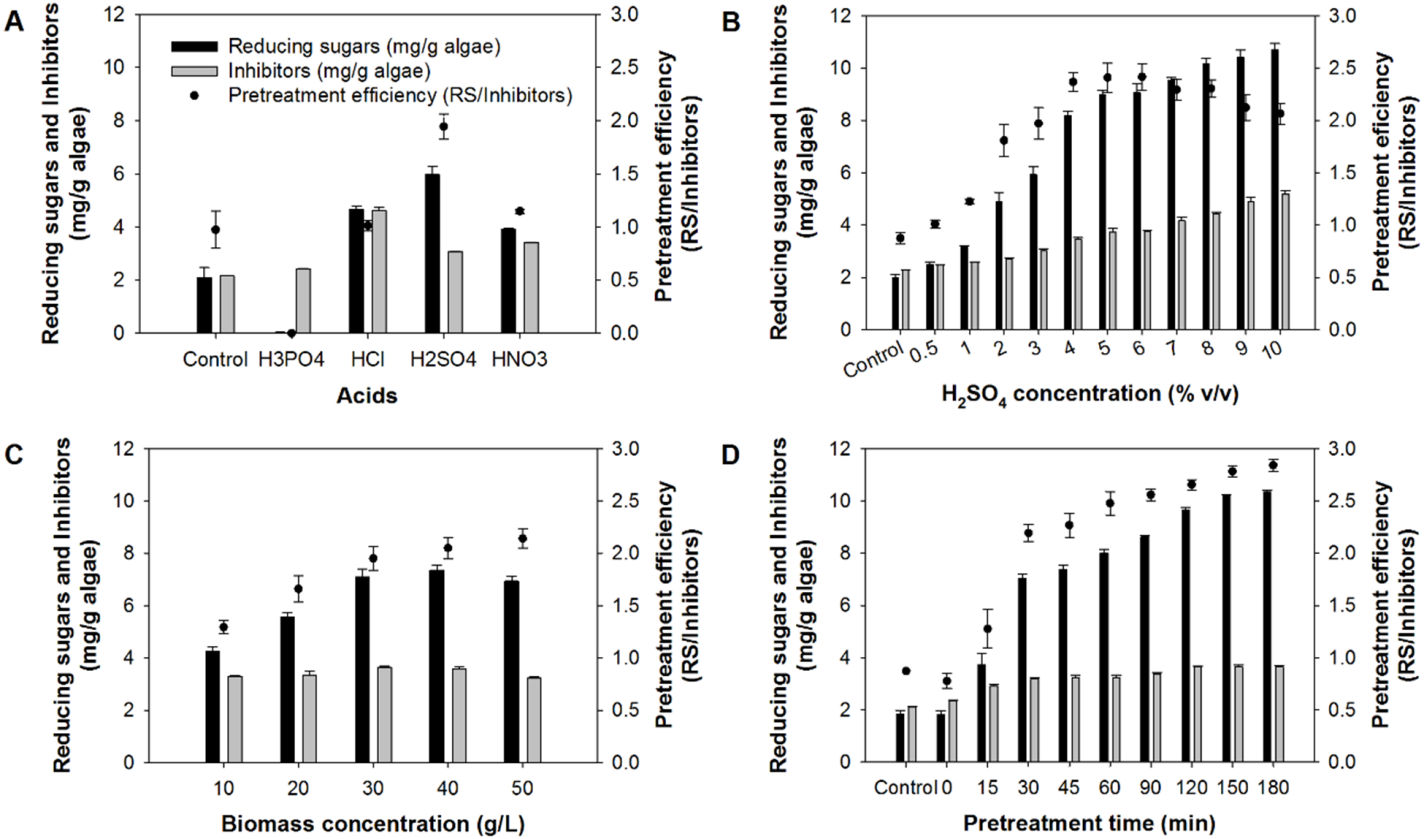
Production of reducing sugars and inhibitors during acid pretreatment of *Chlorella* sp. biomass. (A) Effects of different acids at 3% (v/v) on pretreating 30 g-DW/L of biomass for 30 min. (B) Effects of H_2_SO_4_ concentration on pretreating 30 g-DW/L of biomass for 30 min. (C) Effects of biomass concentration on the pretreatment using 4% (v/v) of H_2_SO_4_ for 30 min. (D) Effects of pretreatment time on pretreating 40 g-DW/L of biomass using 4% (v/v) of H_2_SO_4_ for 30 min. The control was microalgal biomass suspended in distilled water at 35 ± 3 °C.

The aim of pretreating the microalgal biomass was to disrupt the cells and hydrolyze the macromolecules of the cells into readily assimilable nutrients. Among four acids tested, H_2_SO_4_ was found to be the most effective in terms of both sugar production and pretreatment efficiency, followed by HCl and HNO_3_. Almost no reducing sugar was detected in an experiment using H_3_PO_4_ (Fig. 1A). The sugar yield and efficiency obtained with H_2_SO_4_ were two-fold higher than that of the control (5.97 against 2.1 mg/g of reducing sugar, and 1.94 against 0.97 g-reducing-sugar/g-inhibitors). Based on the results, H_2_SO_4_ was selected as the most suitable acid for pretreating *Chlorella* sp. biomass. From Figs. 1B to 1D, it can be seen that increasing acid concentration, biomass concentration, and pretreatment time resulted in higher sugar production. However, the concentration of inhibitors also increased. As a consequence, the use of H_2_SO_4_ at high concentrations, i.e., beyond 6% (v/v), led to lower pretreatment efficiencies (Fig. 1B). Increasing the biomass concentration beyond 40 g/L did not improve the sugar production (Fig. 1C). Instead, at 50 g/L of biomass, the production of reducing sugar slightly decreased. Prolonging the pretreatment time to 180 min resulted in increased production of reducing sugars. Nevertheless, the production of inhibitors was relatively constant when the pretreatment times in a range 30–180 min were used (Fig. 1D). Using the pretreatment time of 180 min, the highest sugar yield of 10.4 ± 0.1 mg/g was attained. Nevertheless, non-significant results (10.2 ± 0.0 mg/g) (*p* = 0.05) was achieved at 150 min. The reducing sugar yield of 10.2 ± 0.0 mg/g was attained under the conditions of 4% (v/v) of H_2_SO_4_, biomass concentration of 40 g/L, and the pretreatment time of 150 min. Considering that a theoretical reducing sugar yield based on the carbohydrate content of *Chlorella* sp. biomass was 324 mg/g, the use of acid pretreatment gave only 3.1% of the theoretical value.

**Figure 2:**
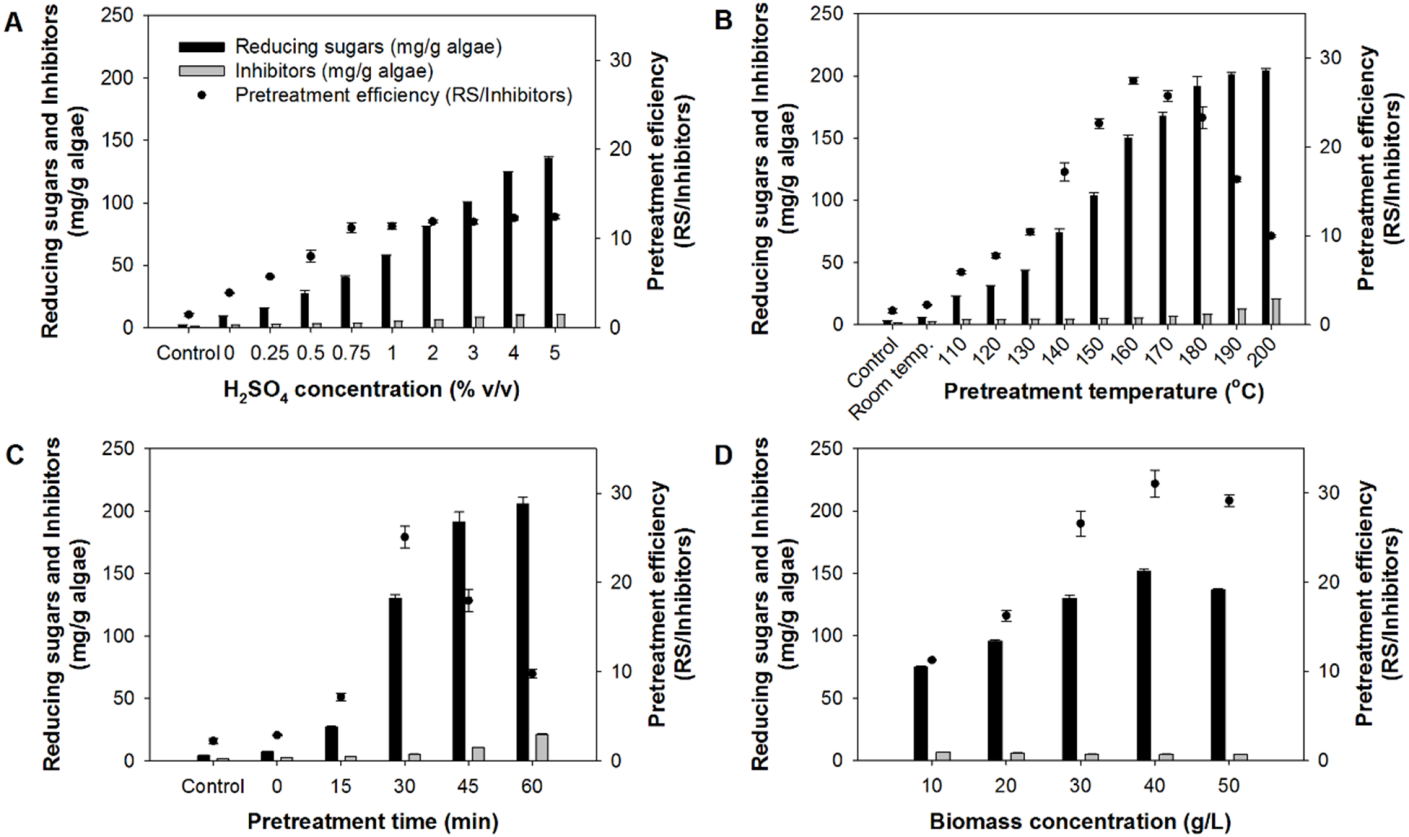
Production of reducing sugars and inhibitors during acid-thermal pretreatment of *Chlorella* sp. biomass. (A) Effects of H_2_SO_4_ concentration on pretreating 30 g-DW/L of biomass at 120 °C for 30 min. (B) Effects of pretreatment temperature on pretreating 30 g-DW/L of biomass using 0.75% (v/v) H_2_SO_4_ for 30 min. (C) Effects of pretreatment time on pretreating 30 g-DW/L of biomass using 0.75% (v/v) at 160 °C for 30 min. (D) Effects of biomass concentration on the pretreatment using 0.75% (v/v) H_2_SO_4_ at 160 °C for 30 min. The control was microalgal biomass suspended in distilled water at 35 ± 3 °C.

The use of acid-thermal method resulted in a reducing sugar yield of 151.8 ± 1.6 mg/g, around 15 times that of the acid method, with the use of lower acid concentration and time. From Fig. 2A, it can be seen that although H_2_SO_4_ concentrations higher than 0.75% (v/v) gave considerably higher sugar production, the formation of inhibitors was also high (Fig. 2A). This could negatively affect hydrogen production. Considering the pretreatment efficiency, 0.75% (v/v) was therefore selected as the optimum. Increasing pretreatment temperature over 160 °C, pretreatment time over 30 min, and biomass concentration over 40 g/L resulted in increased production of inhibitors, leading to lower pretreatment efficiency as can be seen in Figs. 2B–2D, respectively. Overall, the optimum conditions for the acid-thermal pretreatment were 0.75% (v/v) of H_2_SO_4_, pretreatment temperature of 160 °C, pretreatment time of 30 min, and biomass concentration of 40 g/L.

**Figure 3:**
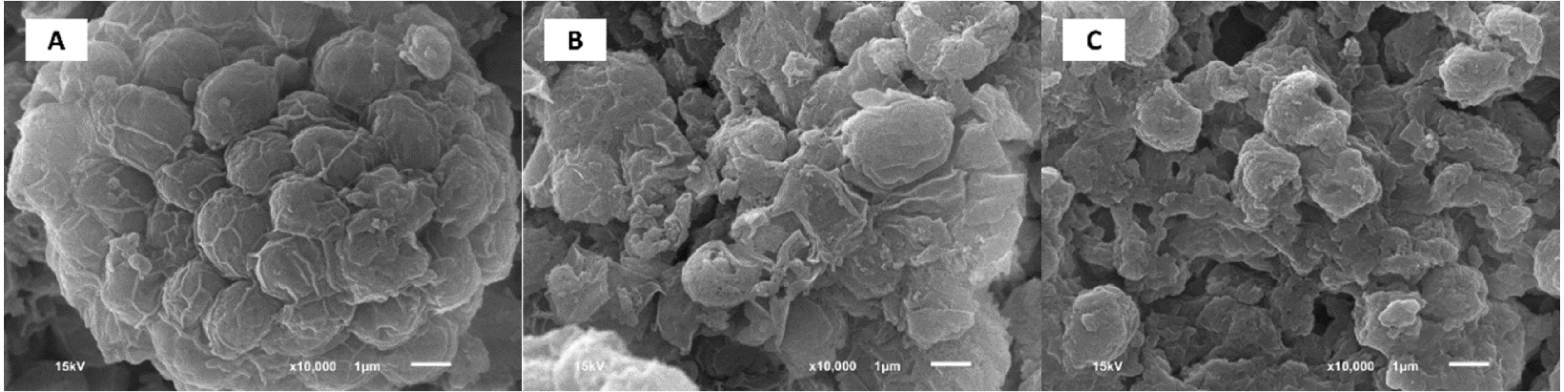
Scanning electron microscope (SEM) images of *Chlorella* sp. biomass at 10,000×. (A) biomass with no pretreatment. Cells were intact with no signs of biomass degradation. (B) biomass pretreated by acid method under the conditions of 4% (v/v) of H_2_SO_4_, biomass concentration of 40 g/L, and the pretreatment time of 150 min. Low degree of cell destruction was observed. (C) biomass pretreated by acid-thermal method under the conditions of 0.75% (v/v) H_2_SO_4_, 160 °C, 30 min, and 40 g-biomass/L. Severe degradation of cells was observed. The bar represents 1 µm.

SEM images of the biomass shown in Fig. 3 confirmed the results of higher reducing sugar production observed in the acid-thermal experiment. Pretreating the biomass with H_2_SO_4_ alone caused only a low degree of destruction (Fig. 3B). On the other hand, the use of H_2_SO_4_ at 160 °C caused more severe degradation (Fig. 3C), which, apart from hydrolyzing macromolecules to smaller units, would help to release the microalgal intracellular components into the hydrolysate, benefitting the subsequent hydrogen fermentation.

### Optimization of hydrogen production from acid and acid-thermal pretreated biomass

**Figure 4:**
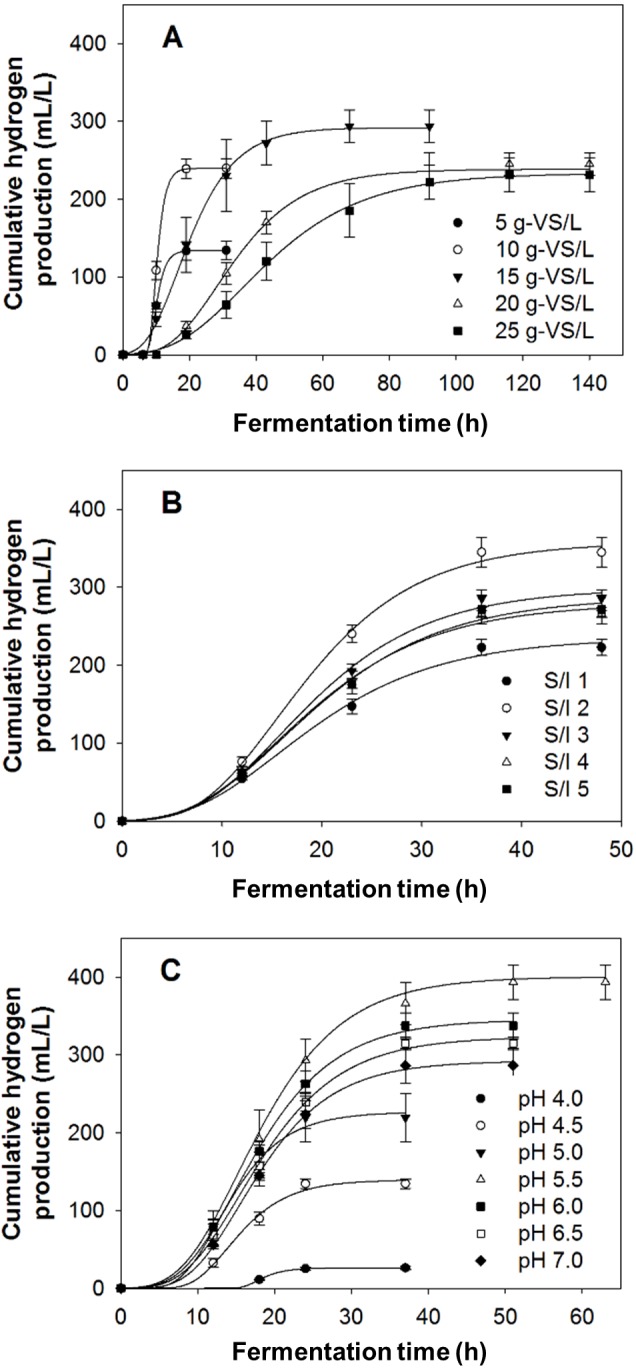
Bio-hydrogen production from acid pretreated *Chlorella* sp. biomass under various conditions. (A) Effect of substrate concentration on hydrogen production using a S/I of 3 and initial pH of 6.0. (B) Effect of S/I ratio on hydrogen production using a substrate concentration of 15 g-VS/L and initial pH of 6.0, and (C) effect of initial pH on hydrogen production using a substrate concentration of 15 g-VS/L and S/I of 2.

The effects of substrate concentration, S/I ratio, and initial pH, on hydrogen production from acid-pretreated biomass are shown in Figs. 4A to 4C. Increasing the substrate concentration from 5 g-VS/L to 15 g-VS/L resulted in increased hydrogen production from 134 ± 12 mL/L to 294 ± 21 mL/L. The production of hydrogen decreased to 231 ± 22 mL/L with further increases in the substrate concentration to 25 g-VS/L (Fig. 4A). Effect of S/I ratio on the production of hydrogen are shown in Fig. 4B. It can be seen that the production of hydrogen was lowest at S/I ratio of 1 (223 ± 10 mL/L), and this increased when S/I ratio was increased to 2. However, further increasing the S/I ratio to 5 resulted in significant decreases in hydrogen production. pH was found to have obvious effect on hydrogen production as seen in Fig. 4C. Increasing initial pH from 4.0 to 5.5 increased the production of hydrogen from 26 ± 2 mL/L to 394 ± 22 mL/L, while further increasing the initial pH to 7.0 led to a decrease in hydrogen production to 287 ± 23 mL/L. Overall, dark fermentation of acid-pretreated biomass under optimum conditions (substrate concentration of 15 g-VS/L, S/I ratio of 2, and initial pH of 5.5) yielded the hydrogen production of 394 ± 22 mL/L, with a yield of 26.3 ± 1.5 mL/g-VS.

**Figure 5:**
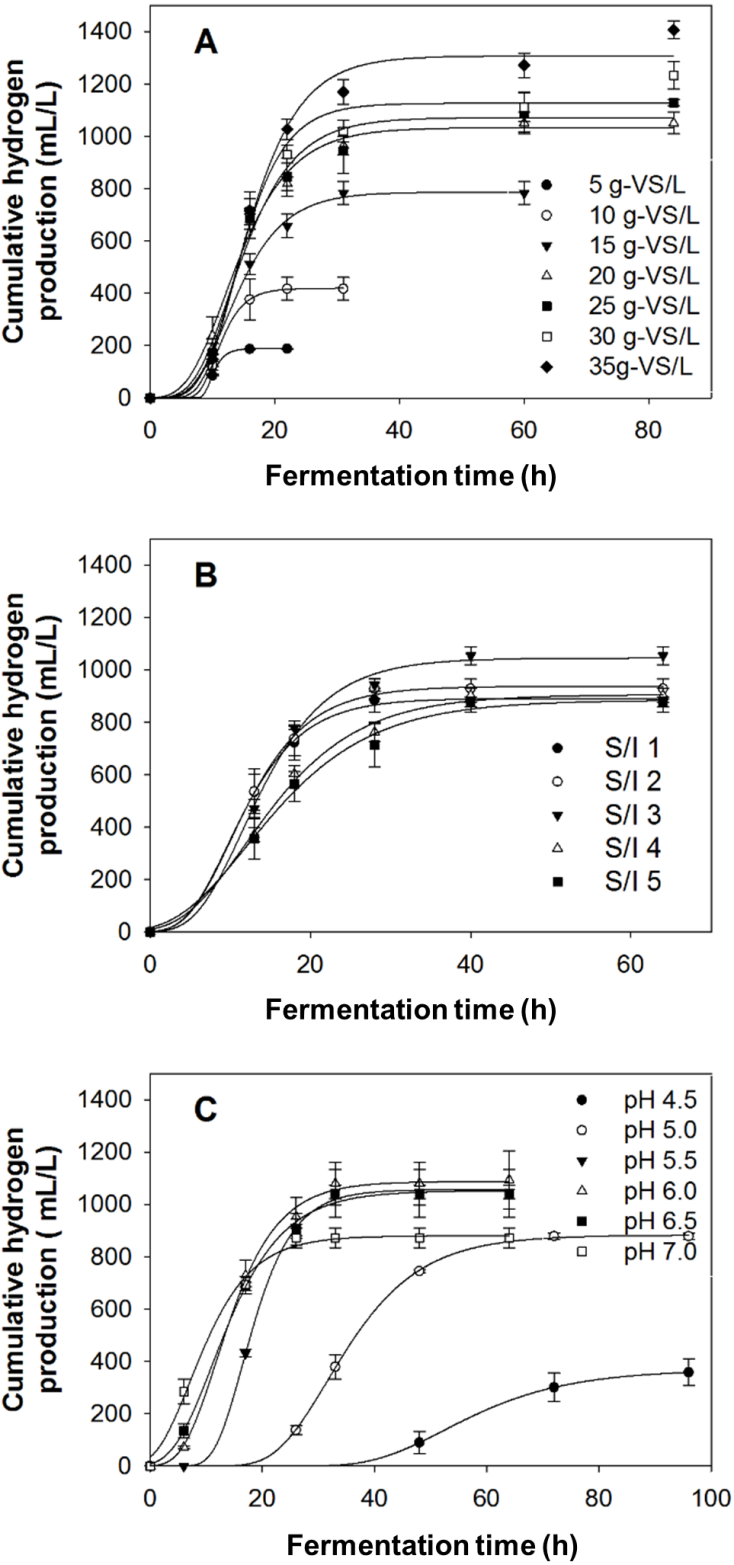
Bio-hydrogen production from acid-thermal pretreated *Chlorella* sp. biomass under various conditions. (A) Effect of substrate concentration on hydrogen production using a S/I of 3 and initial pH of 6.0. (B) Effect of S/I ratio on hydrogen production using a substrate concentration of 25 g-VS/L and initial pH of 6.0, and (C) effect of initial pH on hydrogen production using a substrate concentration of 25 g-VS/L and S/I of 3.

Figure 5 shows effects of substrate concentration, S/I ratio, and initial pH, on hydrogen production from acid-thermal pretreated biomass. From Fig. 5A, it can be seen that hydrogen production increased with increasing substrate concentration from 5 to 35 g-VS/L. However, calculation of HY revealed that it decreased at substrate concentrations above 20 g-VS/L. Hydrogen production was also influenced by S/I ratio as seen in Fig. 5B. Using acid-thermal pretreated biomass, the hydrogen production increased with increasing S/I ratio to 3, then decreased when the ratio was increased to 5. As for the effect of initial pH, Figure 5C shows that the production of hydrogen was highest at the initial pH of 6.0. The use of too low pH values, i.e., pH 4.5–5.5, and too high pH values, i.e., 6.5–7.0, resulted in lower hydrogen production. Overall, the use of acid-thermal pretreated biomass gave 1,079 ± 81 mL/L, with a HY of 54.0 ± 4.1 mL/g-VS, under the optimum conditions of 20 g-VS/L, S/I ratio of 3, and initial pH of 6.0.

### Microbial community during the fermentation of acid and acid-thermal pretreated *Chlorella* sp. biomass

**Figure 6:**
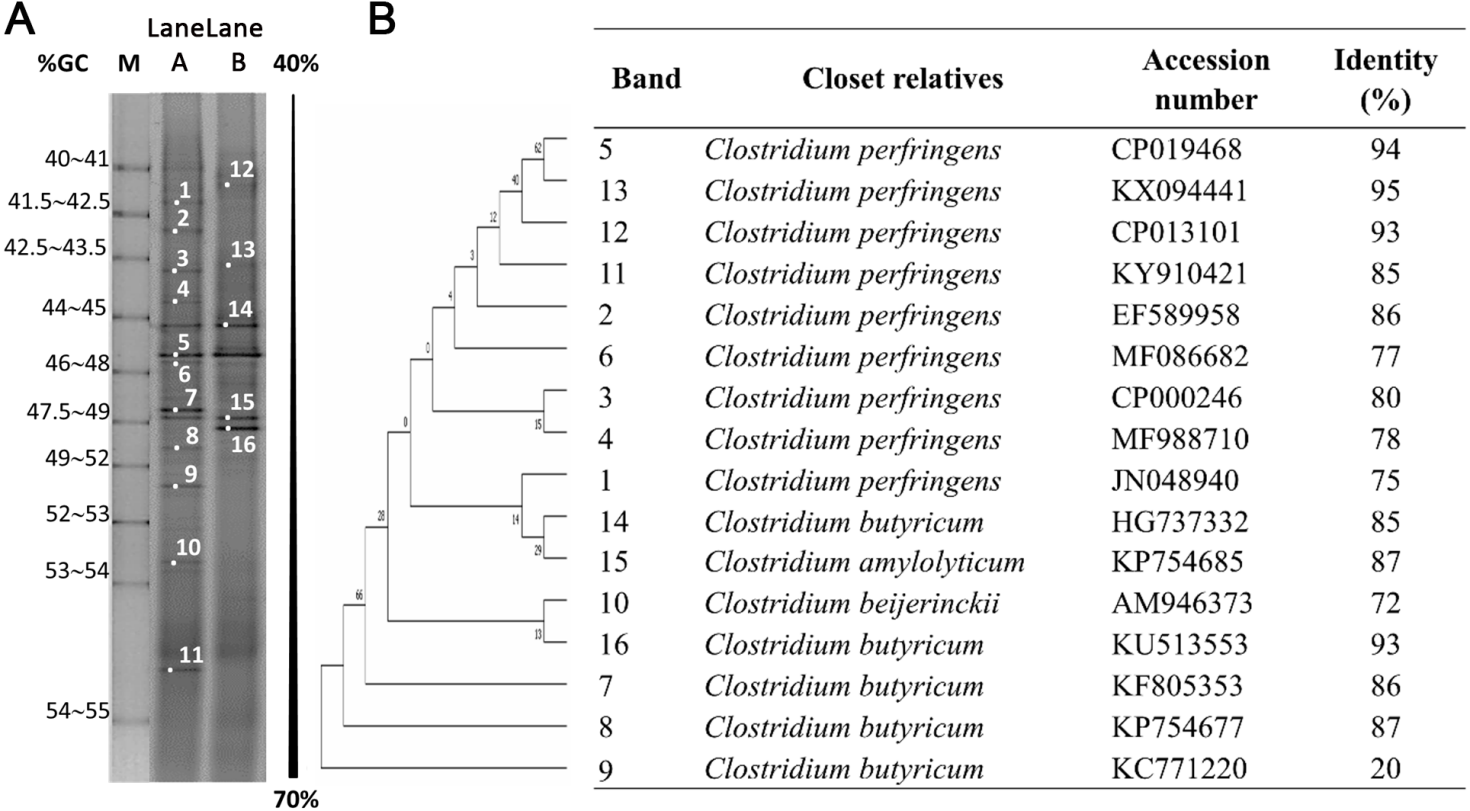
PCR-DGGE profiles of 16S rDNA gene fragments of microbial communities in hydrogen fermentation of acid and acid-thermal pretreated biomass (A), and phylogenetic tree and identity (%) (B). Lane A, acid pretreated biomass; Lane B, acid-thermal pretreated biomass. The identity (%) was calculated by dividing the number of identical nucleotides in the alignment by the length of query sequences.

It is generally known that several factors, e.g., pH and temperature (Kim et al., 2011), substrate concentration (Ning et al., 2013), source of inoculum and inoculum pretreatment method (Ravindran, Adav & Yang, 2010; Cai & Wang, 2016), and phase of microbial growth during the fermentation (*i.e*., lag, exponential or stationary phases) (Fang, Li & Zhang, 2006; Huang et al., 2010) can affect the microbial community, which in turn affect efficiency of hydrogen production process (O-Thong, 2017). Due to the differences in optimum conditions for hydrogen production observed for the acid- and acid-thermal pretreated biomass, it was interesting to investigate whether the microbial community in the two fermentations was different. Using PCR-DGGE, the microbial communities during the late production phase of hydrogen were analyzed. All of the bands shown in Fig. 6A, in both Lanes A (acid pretreatment) and B (acid-thermal pretreatment), showed high identity to *Clostridium* spp. (Fig. 6B). Nevertheless, the different PCR-DGGE profiles indicated that the dominant microbial strains in the two fermentations were different, and this might be the cause of different hydrogen production from the two substrates. From Fig. 6, four bands detected in both experiments with acid and acid–thermal pretreated biomass were affiliated with *Clostridium perfringens* (bands 5 and 6), *C*. *butyricum* (band 14) and *C*. *amylolyticum* (band 15). Nine bands were found only in the fermentation using acid pretreated biomass, which were affiliated with *C*. *perfringens* (bands 1, 2, 3, 4, and 11), *C*. *butyricum* (bands 7, 8, and 9) and *C*. *beijerinckii* (band 10). Three bands affiliated with *C. perfringens* (bands 12 and 13) and *C*. *butyricum* (band 16) were detected only in the acid-thermal hydrolysate fermentation broth.

## Discussion

In acid hydrolysis, H^+^ interacts rapidly with the glycosidic bonds of starch, cellulose, and hemicellulose, resulting in the cleavage of the bonds (Sarip et al., 2016). Therefore, it could be expected that the presence of more H^+^ would lead to a higher degree of hydrolysis. Although based on dissociation constant (K_a_) of the acids, HCl was the strongest acid, followed by H_2_SO_4_, HNO_3_, and H_3_PO_4_, in terms of acid concentration, H_2_SO_4_ had the highest molarity (0.55 M, equivalent to 0.56 M H^+^ after complete dissociation), followed by HCl (0.37 M), HNO_3_ (0.47 M), and H_3_PO_4_ (0.46 M). This could be the reason for the highest reducing sugar production using H_2_SO_4_ as seen in Fig. 1A. It was noticeable that almost no reducing sugar was detected with the use of H_3_PO_4_. This could be possible that H^+^ generated after dissociation of the acid degraded sugars to inhibitors (5-hydroxymethyl furfural and furfural), resulting in less sugar being present in the hydrolysate. As for the effect of H_2_SO_4_ concentration on the pretreatment (Fig. 1B), it was considered that increasing acid concentration from 0.5% (v/v) to 4% (v/v) increased the concentration of H^+^ to a level that was optimum for hydrolyzing the biomass. Further increasing the concentration beyond 6% (v/v) led to lower pretreatment efficiencies, as excessive H^+^ degraded more of the sugar to inhibitors (Yun et al., 2013). Results shown in Fig. 1C revealed that increasing the biomass concentration beyond 40 g/L did not improve the sugar production. This was possibly because high solids concentrations limited the contact between biomass and acid (Park et al., 2016). Effect of high solid loading was also reported by Ho et al. (2013), where enzymatic hydrolysis of *C. vulgaris* FSP-E suspension was hindered at biomass concentrations above 20 g/L. It should be noted that the concentration of biomass at which hydrolysis is limited can be influenced by several factors, including microalgal strain, and operating conditions such as temperature, type of catalyst and its concentration. Effects of pretreatment time on the pretreatment shown in Fig. 1D demonstrated that prolonging pretreatment time to 180 min did not significantly affect the formation of inhibitors although the production of reducing sugar continued to increase. Nevertheless, as mentioned earlier, the reducing sugar yield attained at 150 and 180 min were not significantly different. Therefore, 150 min was selected as the optimum pretreatment time in order to save energy.

The use of acid-thermal method was found to be more effective in pretreating the biomass, compared with the acid method. The highest reducing sugar yield obtained from the acid-thermal method was 151.8 ± 1.6 mg/g, which was much higher than that obtained when acid was used alone (10.2 ± 0.0 mg/g). This was considered due to the synergistic effects of acid and heat in degrading the biomass. This synergistic effect was also reported by Miranda, Passarinho & Gouveia (2012) to be more effective than the use of high temperature alone. It is noteworthy that although the use of acid-thermal method greatly improved the sugar production from the biomass, this method also yielded high inhibitors concentration, as reflected from the decreasing pretreatment efficiency in Figs. 2B to 2D. It is generally known that sugars, particularly glucose and xylose, are dehydrated to HMF and furfural, respectively, at high temperatures, e.g., 120–220 °C (Tsoutsos & Bethanis, 2011; Tan-Soetedjo et al., 2017; Steinbach et al., 2018). Therefore, increasing the pretreatment temperature to above this range could lead to more conversion of sugars into the inhibitors. In the present study, obvious effect of pretreatment temperature on the pretreatment efficiency was observed at 170 °C and higher (Fig. 2B). The profile of reducing sugar as a function of pretreatment time (Fig. 2C) was similar to that of the acid method (Fig. 1D), i.e., the sugar yield increased with increasing time. However, the pretreatment efficiency dropped sharply in the acid-thermal experiment when the pretreatment time was increased to 45 and 60 min. This was considered due to increased exposure time of sugars to acid and heat, which allowed more sugars to be converted into inhibitors. This phenomenon was previously reported in a work of Cao et al. (2009), where increasing H_2_SO_4_ concentration from 0.25% to 4% and pretreatment time from 30 to 180 min caused higher formation of inhibitors, leading to a considerable reduction in hydrogen production from corn stover. As for the effect of biomass concentration on acid-thermal pretreatment of the biomass (Fig. 2D), the biomass concentration had similar effect to that observed in the acid pretreatment experiment. Hydrolysis of the biomass was likely to be hindered at biomass concentration above 40 g/L. Similar explanation for Fig. 1C could be applied.

Effects of substrate concentration, S/I ratio, and initial pH on hydrogen production from acid-pretreated biomass are shown in Figs. 4A to 4C. The use of substrate concentrations of 5 and 10 g-VS/L resulted in low productions of hydrogen (Fig. 4A). It was further noticed that the productions stopped at around 20 h. This signified that assimilable nutrients in the hydrolysates might be completely consumed, and that substrate was provided insufficiently. Generally, increasing substrate concentration and S/I ratio will lead to improved hydrogen production. However, when the concentration of substrate exceeds a certain level, in this case 15 g-VS/L (Fig. 4A) and S/I ratio of 2 (Fig. 4B), hydrogen production decreased. This was possibly due to substrate inhibition and accumulation of the inhibitory substances such as furfural, HMF (Roy et al., 2014), and sodium ion (Na^+^), which was generated as a result of the acid neutralization after pretreatment. With the use of 4% (v/v) H_2_SO_4_, 58 g/L of NaOH was required for neutralization, giving rise to the generation of 33.35 g-Na^+^/L. This was much higher than a level of 5 g/L reported to be inhibitory to microorganisms (Chen, Cheng & Creamer, 2008). The high protein content of the biomass could also contribute to low hydrogen production. With higher concentrations of the biomass, higher generation of ammonia (NH_3_) was expected. pH outside the optimum range can also affect the hydrogen production. Generally, hydrogen production by mixed culture is best at pH 5.5–6.5 (Xia et al., 2016). This was because at low pH values, large amount of H^+^ in the medium can pass through the cell membrane, inhibiting growth of the bacteria and the activity of hydrogenase (Mohan et al., 2013). At high pH values, hydrogen producer shifts the metabolic pathway to solventogenesis, thereby less hydrogen is synthesized (Reungsang & Sreela-or, 2013; Chandrasekhar, Lee & Lee, 2015).

Results shown in Figs. 5A to 5C indicate that substrate concentration, S/I ratio, and initial pH have significant effects on hydrogen production from acid-thermal pretreated biomass. These effects were very similar to those observed for the acid-pretreated biomass. For this reason, the explanation for the acid-pretreated biomass could be applied. It is worth mentioning, based on results reported in Fig. 5A, that although a substrate concentration of 35 g-VS/L gave the highest hydrogen production, 20 g-VS/L gave the highest HY (1,050 ± 42 mL/g-VS). Therefore, from the economical point of view, 20 g-VS/L was selected as the optimum substrate concentration. From the results, it was also noticeable that a higher substrate concentration, and hence higher S/I ratio, could be used when the biomass was previously pretreated using the acid-thermal method. This was possibly because a lower acid concentration was used, compared with the acid method (0.75% against 4%), leading to a lower generation of toxic Na^+^ in the fermentation medium. The detection of acetic acid and butyric acid as the main soluble microbial products in the hydrogenic effluent of both acid- and acid-thermal pretreated biomass, along with traces of propionic acid, formic acid, and lactic acid (Tables S1 and S2), indicated that the fermentation was an acetate-butyrate type.

From Figs. 6A and 6B, only Clostridia were detected in the fermentation systems because the anaerobic granules were heat-treated before use in the fermentations. Heat treatment eliminates vegetative bacteria and methanogens, so that only spore-forming bacteria, e.g., *Clostridium* spp., could thrive during the fermentation. This, coupled with a sampling during the late production phase of hydrogen, was considered the primary reason for the detection of only *Clostridium* spp.

Based on results shown in Figs. 1 to 5, it is clear that acid-thermal method was more effective than the acid method in pretreating biomass of *Chlorella* sp., leading to a higher hydrogen production. It is worth noting that although some recent studies have investigated the use of acid-thermal method to pretreat biomass of microalgae for hydrogen production, for example those of Liu et al. (2012), Ferreira et al. (2013), and Choi et al. (2016), the present study used different microalgal strain, acid, pretreatment conditions, and, more importantly, different approach. This would add further knowledge on microalgal biomass pretreatment, which can consequently contribute to development of hydrogen production from microalgal biomass.

## Conclusions

Pretreating the biomass using an acid-thermal method gave a significantly higher reducing sugar yield compared to acid pretreatment, which led to 174% improvement of hydrogen production. The present study demonstrates clearly that the acid-thermal method is more effective than the acid method in *Chlorella* sp. biomass pretreatment. The study reveals that *Chlorella* sp. biomass is a feasible feedstock for hydrogen production.

##  Supplemental Information

10.7717/peerj.6637/supp-1 Data S1 Reducing sugars, inhibitors, and hydrogen production from acid and acid-thermal pretreatments

10.7717/peerj.6637/supp-2 Table S1 Kinetic parameters for hydrogen production from acid pretreated biomass, and volatile fatty acids in the hydrogenic effluent

10.7717/peerj.6637/supp-3 Table S2 Kinetic parameters for hydrogen production from acid-thermal pretreated biomass, and volatile fatty acids in the hydrogenic effluent

## Additional Information and Declarations

### Data Availability

The following information was supplied regarding data availability: The raw data are available in the Supplemental Files. The raw data file includes the determination of reducing sugar and inhibitors concentrations, as well as the determination of hydrogen production. Tables S1 and S2 show kinetic parameters for hydrogen production, and volatile fatty acids during hydrogen fermentation.
