# Supplementary material for: Improvement of hydrogen production from Chlorella sp. biomass by acid-thermal pretreatment
Source: PeerJ. 2019 Mar 21;7:e6637. doi: 10.7717/peerj.6637 (PMC6431539; doi:10.7717/peerj.6637)
Supplement: Table S2 [file peerj-07-6637-s003.docx]

**Supplemental Table S2**

Estimated kinetic parameters by the use of the modified Gompertz model for hydrogen production, HY and VFAs in the final product of hydrogen production from acid-thermal pretreated *Chlorella* sp. biomass.

| Conditions | *H*_max_  (mL/L) | | HPR  (mL/L·h) | λ (h) | HY  (mL/g-VS) | VFAs (g/L) | | | |
| --- | --- | --- | --- | --- | --- | --- | --- | --- | --- |
|  |  |  |  |  |  | HAc | HBu | HPr | HFo |
| Biomass concentration (g-VS/L) | | | | | | | | | |
| 5 | | 188 | 57.6 | 8.5 | 37.6 | 1.01 | 0.40 | nd | nd |
| 10 | | 419 | 61.7 | 7.9 | 41.7 | 1.80 | 0.72 | 0.13 | nd |
| 15 | | 786 | 56.1 | 6.9 | 52.2 | 2.44 | 0.83 | 0.20 | nd |
| 20 | | 1,032 | 63.8 | 5.9 | 52.5 | 3.31 | 1.08 | 0.35 | nd |
| 25 | | 1,071 | 69.0 | 7.0 | 45.1 | 3.29 | 1.42 | 0.28 | nd |
| 30 | | 1,128 | 89.3 | 8.5 | 41.1 | 2.93 | 1.48 | 0.32 | nd |
| 35 | | 1,406 | 85.4 | 8.1 | 40.2 | 1.58 | 1.84 | 0.18 | 0.09 |
| S/I ratio | | | | | | | | | |
| 1 | | 891 | 63.4 | 4.4 | 44.2 | 3.01 | 0.91 | 0.36 | nd |
| 2 | | 937 | 64.4 | 4.6 | 46.4 | 2.65 | 0.95 | 0.19 | nd |
| 3 | | 1,045 | 65.6 | 5.6 | 52.7 | 2.40 | 1.14 | 0.17 | nd |
| 4 | | 905 | 42.8 | 4.1 | 45.1 | 2.36 | 1.02 | 0.13 | nd |
| 5 | | 884 | 37.5 | 3.3 | 43.9 | 2.49 | 1.28 | 0.23 | nd |
| Initial pH | | | | | | | | | |
| 4.5 | | 369 | 10.9 | 39.9 | 17.9 | 0.68 | 0.86 | nd | 0.05 |
| 5.0 | | 882 | 35.6 | 22.5 | 44.2 | 0.63 | 0.92 | nd | nd |
| 5.5 | | 1,056 | 77.9 | 11.5 | 52.3 | 1.36 | 0.89 | nd | nd |
| 6.0 | | 1,087 | 66.3 | 5.7 | 53.9 | 2.48 | 1.17 | nd | nd |
| 6.5 | | 1,051 | 55.1 | 4.0 | 52.1 | 2.81 | 0.94 | nd | nd |
| 7.0 | | 879 | 53.5 | 1.1 | 43.6 | 2.99 | 0.96 | 0.28 | nd |
| *H*_max_ is maximum hydrogen production, HPR is maximum hydrogen production rate, λ is lag time, HY is hydrogen yield, calculated by dividing the actual maximum hydrogen production by the VS, VFAs is volatile fatty acids, HAc is acetic acid, HBu is butyric acid, HPr is propionic acid, HFo is formic acid, nd = not detected | | | | | | | | | |
